# Minimal invasive vertical hemispherotomy in a 2.5-month-old infant with hemispheric Sturge-Weber Syndrome and recurrent status epilepticus using neuronavigation and augmented reality support

**DOI:** 10.1007/s00381-025-06857-7

**Published:** 2025-05-30

**Authors:** Karl Roessler, Jonathan Weiss, Julia Shawarba, Valerie Quinot, Gregor Kasprian, Martin Niederle, Thomas Czech, Florian Mayer, Christian Dorfer, Martha Feucht

**Affiliations:** 1 Neurosurgical Department, Medical University of Vienna, 1090 Vienna, Austria; 2 Comprehensive Center of Clinical Neurosciences and Mental Health (C3 N-MH), Medical University of Vienna, 1090 Vienna, Austria; 3 Division of Neuropathology and Neurochemistry, Department of Neurology, Medical University of Vienna, 1090 Vienna, Austria; 4 Department of Biomedical Imaging and Image-Guided Therapy, Division of Neuro- and Musculosceletal Radiology, MedUniWien, Vienna, Austria; 5 Department of Anesthesia, Intensive Care Medicine and Pain Medicine, MedUniWien, Vienna, Austria; 6 Department of Pediatrics and Adolescent Medicine, Member of ERN EpiCARE, MedUniWien, Vienna, Austria

**Keywords:** Drug-resistant epilepsy, Sturge-Weber Syndrome, Minimal invasive vertical hemispherotomy, Neuronavigation, Augmented reality

## Abstract

Hemispherotomy in infants under the age of 3 months is considered a high-risk procedure and is not routinely performed. A 2.5-month-old female infant weighing 5.1 kg successfully underwent a right vertical hemispherotomy after developing status epilepticus due to a right hemispheric Sturge-Weber meningo-angiomatosis. The surgical technique involved skull fixation at the skull base using kid pins to facilitate image-guided surgery with neuronavigation and augmented reality within the eyepiece of the microscope employing the vertical hemispherotomy technique. The surgical course was uneventful, with moderate blood loss (100 mL of red blood cells, 90 mL of fresh frozen plasma, and 150 mg of tranexamic acid transfused). The surgery duration was within 2 h. Postoperatively, seizures ceased immediately, and the infant experienced rapid developmental and neurological progress, remaining seizure-free for 8 months after surgery (Engel 1a) now.

## Clinical presentation

We report on a 2.5-month-old female infant born after an uncomplicated pregnancy with an Apgar score of 9/10/10 with port-wine stains on the skin of the left head, face, and body. Familial history was normal. Unfortunately, the newborn started to develop focal clonic and secondary generalized seizures immediately and epileptic spasms with 1 month after birth. The infant was treated with PB, LEV, VGB, and a ketogenic diet without success. An MRI scan 4 days after birth suggested the diagnosis of meningeal angiomatosis involving the whole left hemisphere. This was confirmed by a follow-up MRI 3 weeks later, so that the clinical and radiological reports were consistent with Sturge-Weber Syndrome (SWS). The seizures turned out to be drug resistant and the infant had to be re-hospitalized at the second month of life for seizure control and nutrition by a nasogastral tube.

## Decision for hemispherotomy

**Fig. 1:**
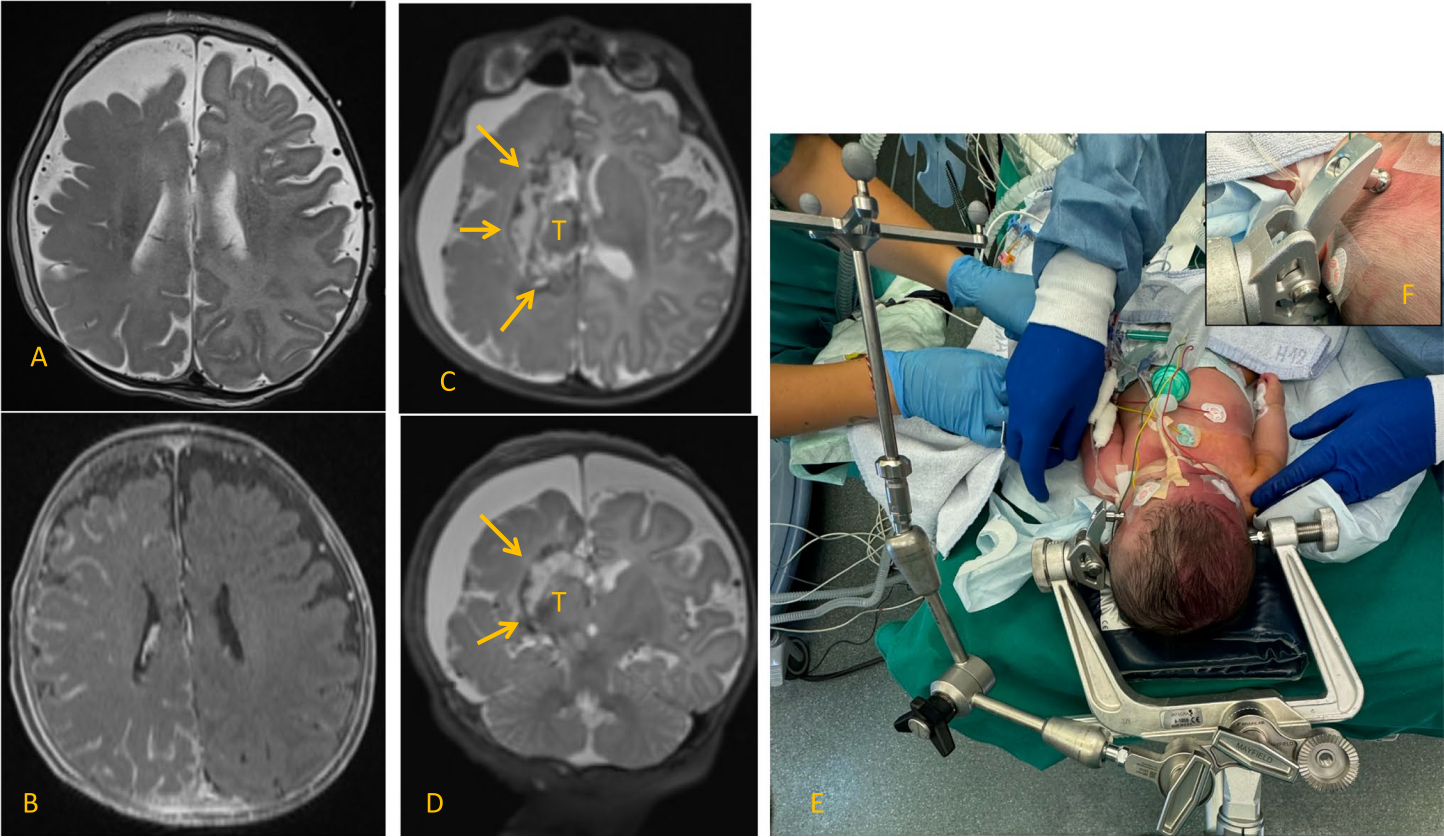
**A** T2 MRI scan showing the developmental delay of the right hemisphere with no gray white differentiation. **B** T1 gadolineum scan with diffuse meningeal enhancement of the complete right hemisphere as the typical appearance of meningeal angiomatosis Sturge-Weber. **C** Postoperative axial T2 MRI scans demonstrating the isolated thalamus on the right side as a result of the minimal invasive functional hemispheric disconnection procedure, arrows demonstrating the disconnection line, T: thalamus. **D** Postoperative coronar T2 MRI scan showing the complete disconnection of the right hemisphere, arrows demonstrating the disconnection line, T: thalamus. **E** Positioning of the infant on a gel pad on the OR table. **F** Head arrested with kids pins on the skull base to overcome the instability of the skull with still open fontanels. Navigation mounted at the skull clamp (Brainlab, Munich, Germany)

Despite 3 different ASM, the seizures could not be attenuated and the infant developed a stuporous state with hemiplegia of the contra-lateral side to the radiological cerebral affection. A status epilepticus was diagnosed and ventilation support and phenobabital drop had to be established at the neonatal intensiv care unit (NICU). Due to the life threatening drug-resistant catastropic epilepsy on the basis of a medically non treatable progressive left hemispheric disease (Sturge-Weber meningeo-angiomatosis, Fig. 1), the surgical option of an early hemispherotomy was discussed in the seizure conference and informed consent obtained from the parents.

## Pro and cons of hemispherotomy in small infants

Due to the small amount of blood volume, there is generally a reluctance of perfoming hemispherotomies in small infants under the age of 3 months and under 6 kg of body weight, equivalent to 500 mL of total blood volume [17, 18]. Additionally, other anesthetic challenges and surgical risks like fragility of the brain, more difficult hemostasis, the need for watertight dural closure, and life threatening perioperative volume and electrolyte imbalances are speaking against early hemispherotomies [10].

Nevertheless, improvements in anesthesia techniques and medication as well as microsurgical progress including technical adjuncts like endoscopy, image-guided surgery using neuronavigation and augmented reality have made the surgery less invasive and much shorter, reducing the blood loss and other reported complications significantly [12, 13].

Additionally, the development of less invasive hemispheric disconnection techniques coming from the traditional hemispherectomy to functional hemispherotomy using the vertical technique might also support the decision for early surgery in very young infants (within the first 3 months and down to 5 kg in weight) if urgently needed [9, 10, 19]. Avoidance of the typical complications have also been extensively adressed within the literature, allowing a more straight forward surgical technique [3].

## Technical aspects of the surgery

**Fig. 2:**
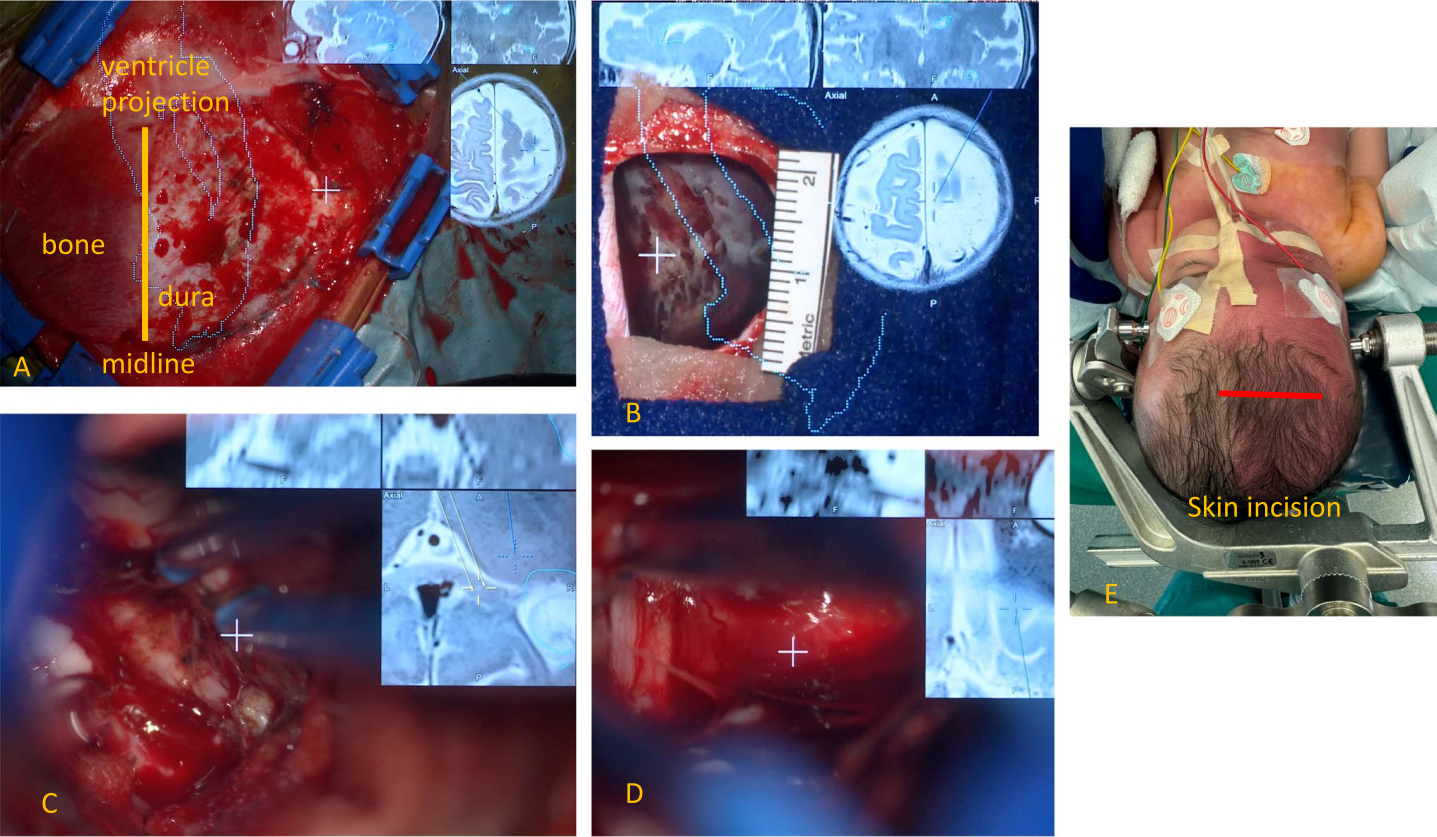
**A** Microscopic view: skin incision (4 cm) parallel to the coronar sutura, 1–2 cm behind the midline, pronounced to the right side. Bone reflected to the midline after lateral craniotomy, with fibrous midline bands as a hinge. 3 plane MRI inserted in the eye piece of the microscope as augmented reality for intraoperative orientation. Contour of the ventricle projected over the operating field. **B** Microscopic view: bone opening measures 2 cm. Typical dark red appearane of the meningeal angiomatosis, like a massive subarachnoid hemorrhage. **C** Microsurgical disconnection of the splenium and **D** Microsurgical disconnection of the frontal lobe along the A1 and M1. **E** Skin incision marked as a red line, note the port-wine stain on the right side of the face. We confirm that we have read the Journal’s position on issues involved in ethical publication and affirm that this report is consistent with those guidelines

For a minimal invasive hemispherotomy, neuronavigation and image guidance are desirable. In this case, the head was put in neutral supine position on a gel plate on the OR table and arrested by a Mayfield clamp mounted on the skull base using kid pins (below 10 lbs pressure, Fig. 2) to be able to use image-guided surgery in a reliable fashion. An electro-magnetic navigation could have been used as an alternative, but by doing so, it would not have been possible to use augmented reality within the microscope because this is not supported by the company.

A coronar skin incision of 5 cm was used 2 cm behind the coronar suture, right-sided pronounced. A part of the right paramedian parietal skull plate was elevated after sawing with the midline bands as a hinge, but not removed. The craniotomy size was 2.5 cm wide. Dural traction stitches were applied at the bone edges to avoid epidural hematomas.

The following procedure was performed using the surgical microscope with introduction of augmented reality and neuronavigational support via the eyepiece of the microscope (Fig. 2).

After dural opening based at the midline, a block of cortex from the superior frontal gyrus down to the ventricle and the corpus callosum removing also the cingulum was resected microsurgically to visualize the ventricle and the corpus callosum. The callosotomy was performed along the interhemispheric fissure, followed by the frontal disconnection. For that, after subpial aspiration of the subcallosal area and posterior gyrus rectus, an incision was made through the floor of the frontal horn and the medial caudate nucleus with the optic nerve, the chiasm, and A1 segment of the anterior cerebral artery as guiding anatomical structures. The perithalamic incision was made with the choroid plexus entering into the temporal horn serving as a guiding structure. We proceeded the resection through the internal capsule by unroofing the temporal horn up to the end of the choroidal plexus at the anterior choroidal point. With the position of the chiasm identified through the fronto-basal disconnection and the optic tract through the opening of the temporal choroidal fissure, connection of these 2 areas along the axis of the optic tract completed the hemispherotomy, illustrated best by the paper from our institution by Dorfer et al. [11] All steps of the hemispherotomy except the interhemispheric callosotomy were performed according to the originally described technique of Delalande et al. [9] Dural closure was performed by watertight stitches and Tachosil (Corza Medical, Vienna, Austria), the bone adapted by non-resorbable stitches. Skin closure was performed using a 4.0 resorbable spiral suture (Monocryl, Ethicon).

No EVD was used during the procedure, postoperative hydrocephalus was avoided by meticulous hemostasis und extensive CSF lavage at the end of the microscopical procedure before closing the dura.

The whole procedure was guided by neuronavigation and augmented reality image guidance, which allowed identification of the anatomical structures much easier and straight forward to save time and blood loss (Fig. 2).

The weight of the reported infant at the time of surgery was 5.1 kg, the body surface 0.31 m [2]. The expected blood volume loss was around 400 mL. During surgery, altogether, 100 mL red blood cells, 90 mL fresh frozen plasma, and 150 mg tranexamic acid were transfused.

## Augmented reality support during the hemispherotomy

The microsurgical procedure was performed using a microscope (Zeiss Kinevo 900, Oberkochen, Germany). This enabled the introduction of augmented reality guidance by preplanning the contours of the ventricles as guidance structures within the Brainlab workstation (Brainlab, Munich, Germany). The microscope served as a navigation tool, projecting the ventricles’ contours onto the surgical field. Additionally, the suction tip was navigated to be visualized in three planes within the microscope’s eyepiece, which functioned as an augmented reality head-up display. Furthermore, the suction tip acted as a pointer device, allowing for the correlation of anatomical structures in the surgical field with the structures on the implemented three-plane MRI scans within the microscope’s eyepiece. This enabled a detailed anatomical understanding of the structures that needed to be disconnected during the surgery, which could be achieved by continuously observing the surgical field through the microscope (Fig. 2).

## Postoperative course

The postoperative course was uneventful, the seizures stopped immediately after surgery, and no fluid or electrolyte inbalances occured. Neurologically, the girl presented only a very mild hemiparesis, the nasogastral tube could be removed, and bottle feeding was possible immediately. Altogether, the infant experienced a steep developmental and neurological progress and could be tranfered to rehabilitation center 2 weeks after surgery. Eight months after surgery, the child is still seizure free since surgery (Engel 1a).

## Literature review

In Table 1, the most important literature about hemispherotomies in infants is summarized. Only in the multicenter retrspective study of Roth et al. [18], the case report of Makridis et al. [16], and the review of Makridis et al. [15], the youngest infants having had a hemispherotomy were as young as our descibed case. The other authors reporting about minimal inveasive techniques for hemispherotomy in infants like endoscopy, thermo-coagulation, robotic guidance, or burr hole technique operated all on older infants. Authors describing larger recent series of hemispherotomies in infants had their youngest patients from 4 to 36 months.

**Table 1:** Literature about hemispherotomy age in infants

Author	Topic	Youngest patient
Cook et al., 2004 [8]	Cerebral hemispherectomy in pediatric patients with epilepsy: comparison of three techniques by pathological substrate in 115 patients	24 months
Chandra et al., 2015 [5]	Endoscopy-assisted interhemispheric transcallosal hemispherotomy in children	4 months
Chandra et al., 2019 [6]	Endoscope-assisted (with robotic guidance and using a hybrid technique) interhemispheric transcallosal hemispherotomy	36 months
Makridis et al., 2021 [16]	Case report: hemispherotomy in the first days of life to treat drug-resistant lesional epilepsy	42 days
Chandra et al., 2021 [4]	A new “bloodless” technique for minimally invasive robotic thermocoagulative hemispherotomy (ROTCH) in children	36 months
Roth et al., 2021 [18]	Epilepsy surgery in infants up to 3 months of age: safety, feasibility, and outcomes: a multicenter, multinational study	2 months
Makridis et al., 2022 [14]	Epilepsy surgery in the first 6 months of life: a systematic review and meta-analysis	Not applicable
Makridis et al., 2023 [15]	Epilepsy surgery in early infancy: a retrospective, multicenter study	83 days
Cicutti et al., 2024 [7]	Surgical anatomy and technique of peri-insular hemispherotomy in pediatric epilepsy	< 12 months
Bergman et al., 2024 [2]	Hippocampal resection during hemispherotomy: is it needed?	24 months
Villamil et al., 2024 [20]	Pediatric peri-insular hemispherotomy and functional hemispherectomy for severe medically refractory epilepsy: comparison of two techniques	60 months mean
Baumgarten et al., 2025 [1]	Burr hole hemispherotomy: case series	18 months
Zheng et al., 2025 [21]	Outcomes and complications of vertical parasagittal hemispherotomy in children: a nationwide population-based study	22 months

## Conclusion

Vertical hemispherotomy in very small infants augmented by image guidance and augmented reality might be a less invasive surgical option compared to standard hemispherotomy techniques to avoid long lasting surgeries and high amount of blood loss.

## Data Availability

No datasets were generated or analysed during the current study.

## Abbreviations

ASM: Anti-seizure medication
PB: Phenobarbital
LEV: Levitiracetam
VGB: Vigabatrin
MRI: Magnetic resonance tomography
NICU: Neonatal intensive care unit
OR: Operating room
SWS: Sturge-Weber Syndrome
